# Conversion of Barnett continent reservoir to Kock reservoir: A 30-year retrospective study on surgical outcomes and long-term follow-up

**DOI:** 10.1007/s10151-026-03310-y

**Published:** 2026-03-27

**Authors:** A. Alipouriani, L. C. Duraes, D. Liska, S. D. Holubar, S. R. Steele, O. Lavryk

**Affiliations:** Department of Colorectal Surgery, Digestive Disease Institute, Cleveland Clinic Foundation, Cleveland Clinic Main Campus, 9500 Euclid Ave, Cleveland, OH 44122 USA

**Keywords:** Barnett continent reservoir, Kock pouch, Continent ileostomy, Pouch salvage, Pouch failure, Surgical conversion, Revision surgery, Long-term outcomes

## Abstract

**Background:**

The Barnett continent reservoir has long been used as a surgical option for patients requiring continent ileostomies, but complications such as valve leakage, obstruction, and pouch failure often necessitate revisions. Conversion to a Kock (K) pouch offers an alternative with potentially improved outcomes. This study evaluated the safety (postoperative complications), feasibility (technical completion of conversion), and effectiveness (long-term pouch salvage) of conversion.

**Methods:**

We conducted a retrospective cohort study of patients who underwent Barnett-to-K-pouch conversion (conversion group) or remained with a Barnett pouch (non-conversion group) between 1991 and April 2024. The conversion technique included disconnection of the afferent limb, preservation and 180° rotation of the reservoir, resection of the collar valve, creation of a stapled nipple valve, and pouch–neo-afferent limb anastomosis. Demographics, surgical history, and long-term follow-up were collected. Pouch salvage was defined as retention of a functional continent pouch; failure was defined as excision with permanent ileostomy.

**Results:**

Out of 30 patients with Barnett pouch patients included, 16 underwent conversion and 14 did not. Median age was 51.5 versus 60.5 years, and median body mass index (BMI) was 24.7 versus 22.3 kg/m^2^ in conversion versus non-conversion groups, respectively. Ulcerative colitis was the primary indication (62.6% versus 78.8%). Leading revision causes were valve slippage (50% versus 35.7%) and fistula (18.7% versus 28.6%). Pouch salvage was higher with conversion (81.3%, 95% confidence interval [CI] 54–96%) versus non-conversion (35.7%, 95% CI 13–65%; *p* = 0.03). Long-term follow-up (median: 159.5 versus 301.6 months,) showed that 60% of all patients in conversion group retained continent pouches, while the remaining experienced pouch excision, indicating an overall failure rate of 40%.

**Conclusions:**

Conversion of Barnett to K-pouch improves pouch salvage and reduces failure. While technically complex, the procedure can be safely offered to motivated patients seeking to avoid permanent ileostomy.

## Introduction

The Barnett continent reservoir (BCR) was introduced in the 1980s as a modification of the earlier continent ileostomy concept originally developed by Dr. Nils Kock [1, 2]. Kock’s design, often referred to simply as the Kock pouch, uses an intussuscepted nipple valve to maintain continence; an external catheter is intermittently inserted through a small stoma to empty the reservoir. By eliminating the external appliance required in a conventional ileostomy, the Kock pouch was a major advancement in quality of life for patients who needed an ileostomy but desired a more discreet and continent option [1, 3].

The Barnett continent reservoir retained an intussuscepted nipple valve but further reinforced it with an external “collar” of ileum, designed to provide additional support and continence [2, 4]. The collar valve was intended to reduce valve‐related problems, such as slippage or leakage, by wrapping and securing a segment of the ileum around the pouch inlet to create a continence zone. However, over time, it became apparent that BCRs continue to experience a variety of complications, including the very issues they sought to address: valve slippage, fistula formation (particularly around the valve), stricture at the reservoir inlet, and occasional complete pouch failure [5]. Once the collar valve loses its integrity, patients often develop chronic leakage or difficulty with reservoir emptying, leading to repeated revisions and hospitalizations. If these measures fail, patients may require pouch excision and transition to a permanent end ileostomy, a step many find psychologically difficult [5, 6].

In contrast, the Kock pouch’s original method of creating a nipple valve by intussuscepting the bowel onto itself generally offers a stable, well‐anchored continence mechanism that, with proper surgical technique, can remain reliable for many years. When BCRs fail, surgeons can repair the pouch or convert the existing reservoir into a Kock pouch by reconstructing the valve portion. While such conversions have been reported to restore continence and avoid permanent ileostomy, relatively few large or long‐term outcome studies exist to quantify their success [5, 7]. Understanding the durability of the neo-valve, the rates of postoperative complications, and overall pouch salvage is vital for surgeons and patients deciding between repeated Barnett revisions or definitive conversion to a Kock pouch.

The present study addresses this knowledge gap by retrospectively examining a 30‐year experience of Barnett reservoir failures managed either by conversion to a Kock pouch or non‐conversion approaches. We compare rates of pouch salvage, the frequency of persistent or recurrent complications, and ultimately the proportion of patients who avoid permanent ileostomy. By focusing on long‐term follow‐up in a sizable cohort, our aim is to offer a clearer understanding of the safety and effectiveness of the Kock pouch conversion technique for individuals with a failing BCR.

## Methods

### Study design and setting

This study was conducted as a retrospective cohort review over a 30-year period, from 1991 to April 2024. All data were collected from patients treated at a single institution. Patient information was gathered through detailed chart reviews and clinical follow-up visits, providing a comprehensive view of outcomes.

### Patient population

The study population included patients with a previously constructed Barnett pouch who had undergone at least one revision owing to pouch complications. Patients were divided into two groups: those who underwent surgical conversion of their Barnett pouch to a Kock pouch (conversion group) and those who did not undergo conversion and were managed with conservative measures or repeated revisions of their Barnett pouch (non-conversion group). Patients were considered for conversion on the basis of preoperative counseling and intraoperative assessment. Before surgery, patients were counseled about the possibility of pouch preservation with conversion versus the need for permanent end ileostomy if conversion was not feasible. Intraoperative findings often dictated the final decision. Conversion was not pursued in cases with insufficient small bowel length (typically < 250 cm) to construct a new valve or in the presence of severely dilated proximal bowel with prestenotic dilation.

### Surgical details

**Fig. 1:**
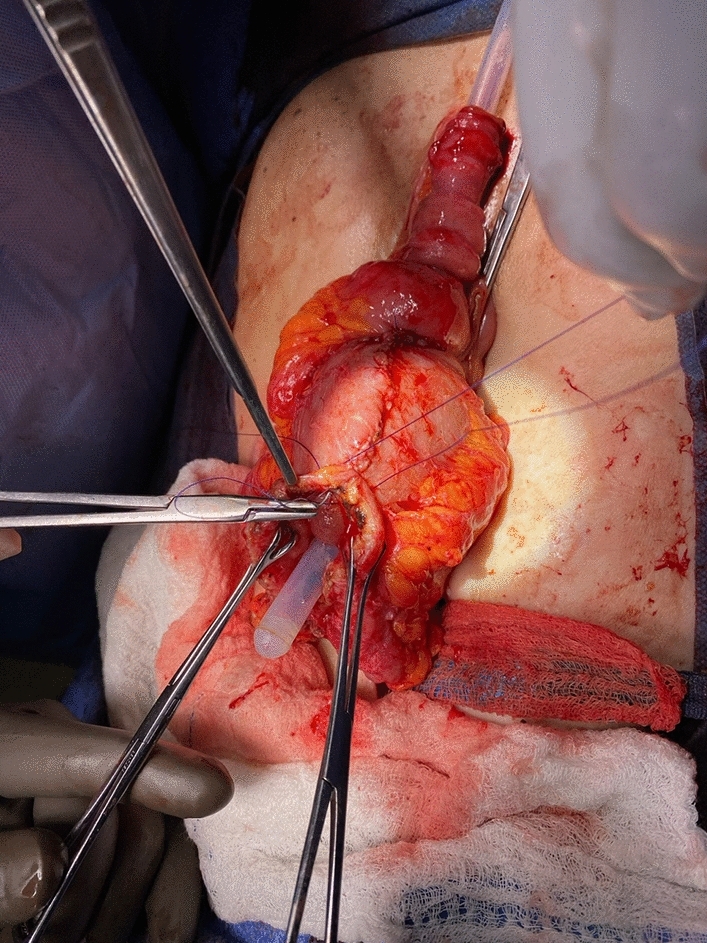
Revision of K pouch with creation of new nipple valve with pouch intubation

All Barnett pouches had been initially constructed at outside institutions. The surgical conversion from a Barnett pouch to a Kock pouch followed a standardized approach. First, the afferent limb was disconnected from the existing Barnett reservoir. The reservoir itself was rotated 180 degrees to ensure proper bowel orientation. The collar valve, which is characteristic of the Barnett pouch, was resected and replaced with a newly constructed nipple valve (Fig. 1).

**Fig. 2:**
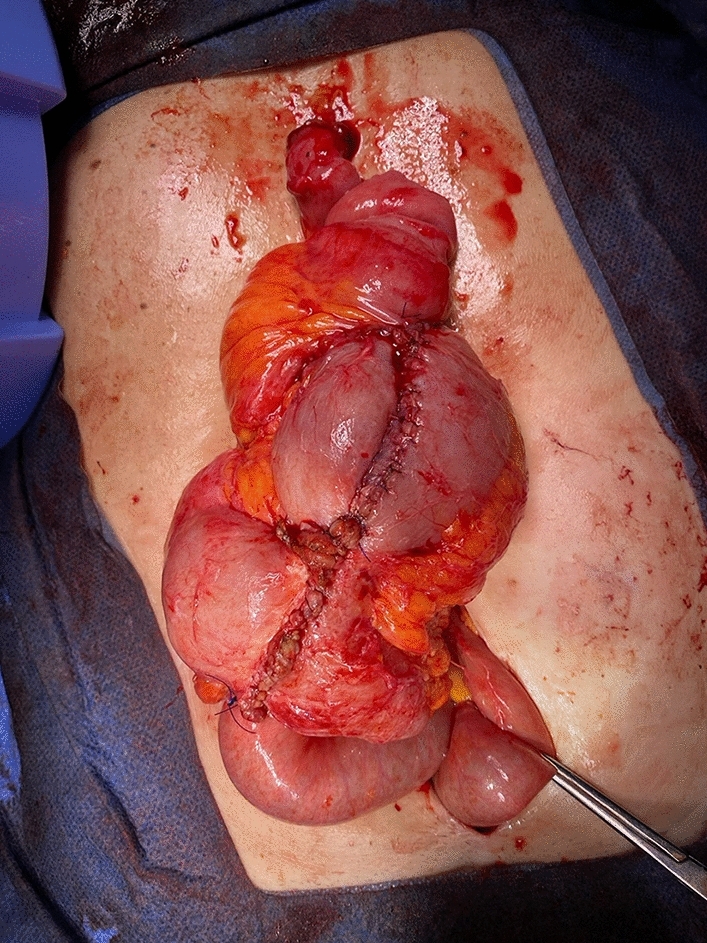
Finalized appearance of K pouch with new nipple valve and hand-sewn closure of pouch enterotomy

**Fig. 3:**
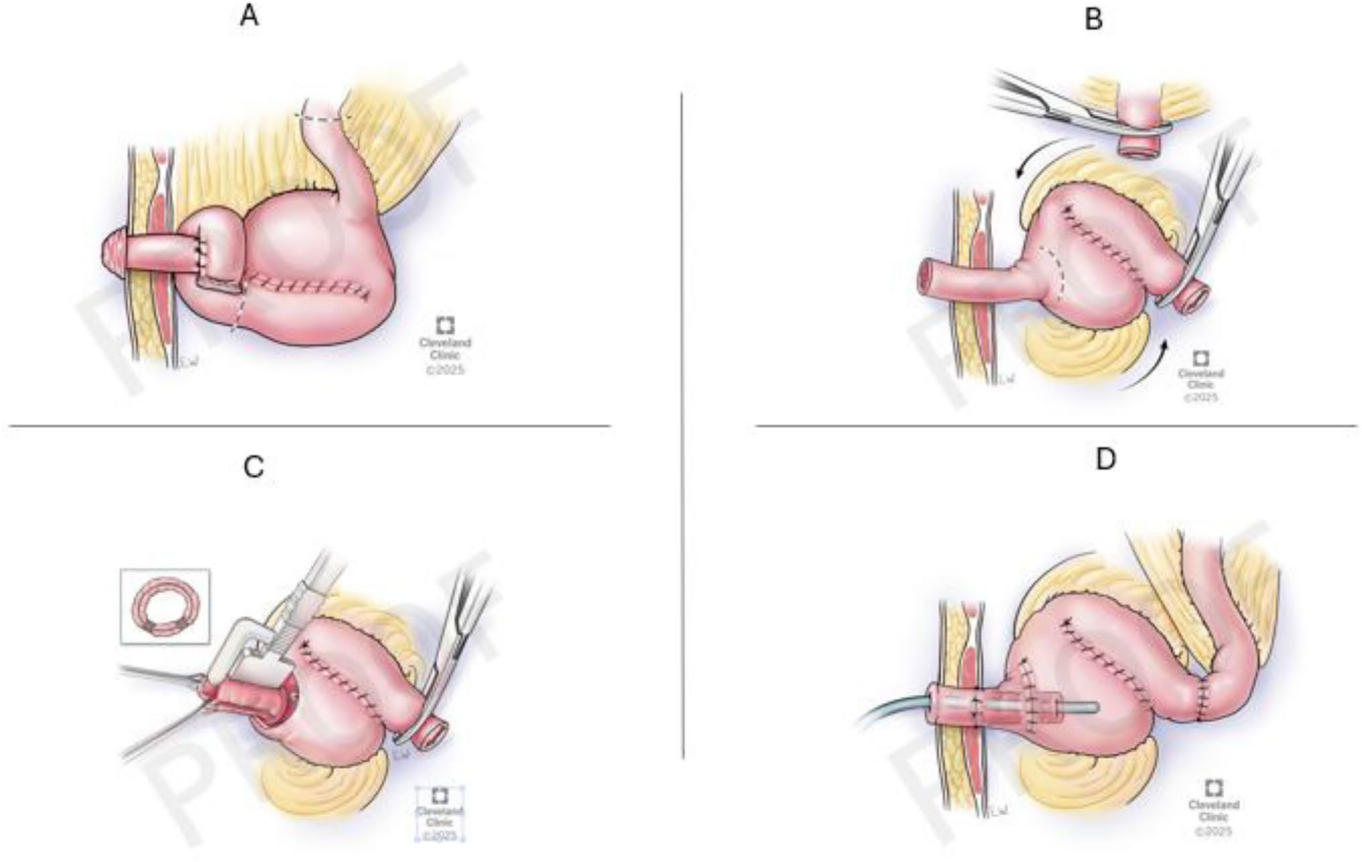
Step-by-step surgical conversion of Barnett continent reservoir to Kock pouch. **A** Resection of the malfunctioning collar valve from the original Barnett reservoir. **B** Rotation of the preserved ileal reservoir by 180 degrees to reorient the bowel for nipple valve construction. **C** Construction of the nipple valve using a 12-cm segment of ileum, invaginated and secured with three non-cutting TA stapler fires, followed by a hand-sewn anastomosis to the new afferent limb. **D** Final appearance of the converted Kock pouch with intact nipple valve and functional continent stoma

This new valve was created by intussuscepting approximately 12 cm of ileum and securing the invaginated segment using three fires of a non-cutting TA stapler. Finally, a hand-sewn end-to-end anastomosis was performed between the preserved reservoir pouch and the newly created afferent limb (Fig. 2 Fig. 3). Throughout this process, the walls of the original Barnett reservoir were preserved, and only the malfunctioning collar valve was replaced with the new valve mechanism [1, 2]. All conversions were performed at our institution by six colorectal surgeons experienced in continent ileostomy reconstruction.

### Data collection

Demographic data, including age, sex, and body mass index (BMI), were collected from the medical records. Clinical variables such as the primary diagnosis (e.g., ulcerative colitis or Crohn’s disease) and indications for the initial surgery were documented. The reasons for revisions, including slipped valve, fistula, bowel obstruction, and stricture, were also recorded. For patients in the conversion group, operative details, including the duration of surgery, length of hospital stay, and postoperative complications, were extracted from the medical records. Long-term outcomes were categorized as either pouch salvage or pouch failure. Pouch salvage was defined as the maintenance of a functional, continent pouch, while pouch failure was defined as the excision of the pouch and the creation of a permanent end ileostomy. Functional outcomes, such as continence scores or quality-of-life measures, were not available in this dataset.

### Statistical analysis

Statistical analyses were conducted using R version 4.3.1. Continuous variables, such as age, BMI, and follow-up time, were summarized as medians with interquartile ranges (IQRs). Categorical variables, including sex distribution, primary diagnosis, revision indications, and final pouch status, were reported as frequencies and percentages. Comparisons between the conversion and non-conversion groups were performed using chi-squared tests for categorical variables. For continuous variables, *t*-tests or their nonparametric equivalents were applied. Statistical significance was defined as a *p*-value less than 0.05. All analyses were performed in R version 4.3.1 to ensure reproducibility and consistency in statistical evaluation.

## Results

### Patient demographics

A total of 30 patients with a Barnett pouch were included in the study, of whom 16 underwent conversion to a Kock pouch (conversion group), and 14 did not (non-conversion group). The median age in the conversion group was 51.5 years (interquartile range [IQR]: 46.5–62.2 years), while in the non-conversion group, it was 60.5 years (IQR: 53–65 years), with no statistically significant difference between the groups (*P* = 0.3). Females comprised 62.5% of the conversion group and 57.1% of the non-conversion group, showing no significant difference between the two groups. The median BMI was also comparable between the groups, being 24.7 (IQR: 23.1–27.6) kg/m^2^ in the conversion group and 22.3 (IQR: 21.4–24.4) kg/m^2^ in the non-conversion group (*p* = 0.7). The most common primary surgical indication for both groups was ulcerative colitis, accounting for 62.6% in the conversion group and 78.6% in the non-conversion group (*p* = 0.5). Crohn’s disease was the second most frequent indication, occurring in 25% of the conversion group and 14.3% of the non-conversion group (*p* = 0.7). One patient in each group had familial adenomatous polyposis (6.2% versus 7.1%), and one patient in the conversion group had a diagnosis categorized as “other” (6.2%), which included acute perforation, pouch fecalith, or polyposis (Table 1).

**Table 1:** Demographics and follow-up

	Converted *N* = 16	Non-converted *N* = 14	*p*-value
Age^1^, years	51.5 (46.5–62.2)	60.5 (53–65)	0.3
Sex, female	10 (62.5%)	8 (57.1%)	
BMI, kg/m^2^	24.7 (23.1–27.6)	22.3 (21.4–24.4)	0.7
Surgical indication disease Ulcerative colitis Crohn’s disease Familial adenomatosis polyposis Other^2^	10 (62.6%) 4 (25%) 1 (6.2%) 1 (6.2%)	11 (78.6%) 2 (14.3%) 1 (7.1%) 0 (0%)	0.5 0.7 1 1
Revision indication Slipped valve Fistula Stricture Recurrent bowel obstructions Crohn’s disease Other^3^	8 (50%) 3 (18.7%) 3 (18.7%) 2 (12.5%) 1 (6.2%) 1 (6.2%)	5 (35.7%) 4 (28.6%) 3 (21.4%) 2 (14.3%) 0 0	0.6 0.8 1.0 1.0 1.0 1.0
Initial Barnett pouch construction outside of the hospital	16 (100%)	14 (100%)	
Median time from Barnett pouch construction to the first revision, months	131.3 (109.7–213.5)	254.8 (106–312.2)	0.0009
*Long-term follow-up* Current pouch status Continent Removed	13 (81.2%) 3 (18.8%)	5 (35.7%) 9 (64.3%)	**0.03** **0.03**
Median follow-up, months	159.5 (132.1–291.5)	301.6 (146.4–652.2)	0.4

Significant difference defined if *p*<0.05

^1^At time of first revision

^2^Acute perforation, pouch fecalith, polyposis

^3^Gastrointestinal dysmotility due to spinal injury

### Revision indications

The leading indications for revision surgery in both groups were valve slippage and fistula formation. In the conversion group, valve slippage occurred in 50% of patients, compared with 35.7% in the non-conversion group (*p* = 0.6). Fistulas were present in 18.7% of patients in the conversion group and 28.6% in the non-conversion group (*p* = 0.8). Strictures were equally common, affecting 18.7% of the conversion group and 21.4% of the non-conversion group (*p* = 1.0). Recurrent bowel obstructions were observed in 12.5% of the conversion group and 14.3% of the non-conversion group (*p* = 1.0). Crohn’s disease recurrence as a reason for revision occurred in one patient in the conversion group (6.2%) but was not observed in the non-conversion group. Other revision indications, such as gastrointestinal dysmotility due to spinal injury, were reported in 6.2% of the conversion group but not in the non-conversion group.

### Time intervals

The median time from Barnett pouch construction to the first revision was significantly shorter in the conversion group compared with the non-conversion group. Patients in the conversion group underwent their first revision after a median of 131.3 (IQR: 109.7–213.5) months, while in the non-conversion group, the time to the first revision was 254.8 (IQR: 106–312.2) months (*p* = 0.0009). For patients in the conversion group, the median time from the initial Barnett pouch construction to Kock pouch conversion was 328.3 (IQR: 274.4–533.8) months (Table 2).

**Table 2:** Details of the patient who underwent conversion to a K-pouch (*n* = 16)

Variables	
Median Time from Barnett pouch construction to K-pouch, months	328.3 (274.4–533.8)
Intraoperative information in patients who converted to K-pouch: Length of stay in hospital, days Surgery duration, minutes	6 (5–8.4) 242 (154–279)
Complications after conversion to K-pouch (*n* = 16) Fistula Incontinence Stenosis No complication Follow-up (months), median	2 (12.5%) 2 (12.5%) 1 (6.2%) 12 (75%) 31.5 (4.7–126.5)

### Outcomes

Long-term follow-up revealed significant differences in pouch salvage and failure rates between the two groups. In the conversion group, 81.3% (13/16; 95% CI, 54.4–95.9%) of patients retained a functional, continent pouch, compared with 35.7% (5/14; 95% CI, 12.8–64.9%) in the non-conversion group (*p* = 0.03). Conversely, pouch failure, defined as the need for pouch excision and creation of a permanent end ileostomy, occurred in 18.7% (3/16) of patients in the conversion group, compared with 64.3% (9/14) in the non-conversion group (*p* = 0.03). The overall median follow-up time was 159.5 (IQR: 132.1–291.5) months for the conversion group and 301.6 (IQR: 146.4–652.2) months for the non-conversion group, with no statistically significant difference between the groups (*p* = 0.4). Across both groups, 60% of all patients retained a continent pouch, while the remaining 40% experienced pouch failure (Table 1***).*** In an exploratory multivariable logistic regression adjusting for age, sex, and diagnosis, conversion remained associated with improved pouch salvage (OR 14.4, 95% CI 1.3–158.5, *p* = 0.03).

### Complications after conversion

Among the 16 patients who underwent conversion to a Kock pouch, postoperative complications were infrequent. Fistula formation and incontinence were each observed in 12.5% (2/16) of patients, while stenosis occurred in 6.2% (1/16). The majority of patients (75%, 12/16) experienced no postoperative complications following the conversion. The median hospital stay for patients undergoing conversion was 6 (IQR: 5–8.4) days, and the median operative time was 242 (IQR: 154–279) min. The median follow-up time after conversion to a Kock pouch was 31.5 (IQR: 4.7–126.5) months (Table 2).

## Discussion

This study provides important insights into the outcomes of patients who underwent conversion of a failing Barnett pouch to a Kock pouch, as well as those managed without conversion. The principal finding is the significantly higher pouch salvage rate in the conversion group, with 81.3% of patients retaining a functional, continent pouch compared with only 35.7% in the non-conversion group. Similarly, pouch failure, defined as the need for excision and creation of a permanent end ileostomy, was markedly lower in the conversion group at 18.7%, compared with 64.3% in the non-conversion group. These findings underscore the efficacy of Kock pouch conversion in salvaging failed Barnett reservoirs, suggesting that it provides a durable solution for patients who otherwise face the psychological and physical challenges of permanent ileostomy.

When comparing these results to prior literature, it becomes evident that this study offers a unique contribution through its long-term follow-up and focused evaluation of Barnett-to-Kock pouch conversions. Earlier reports on Barnett reservoirs and Kock pouches highlighted the potential for continent ileostomy to improve patient quality of life [1, 2]. However, they also documented complications, including valve slippage, fistula formation, and pouch failure, which remain significant challenges in these procedures. While both designs were pivotal in advancing the field of continent ileostomy, long-term comparative outcomes between the two techniques have been limited. This study’s retrospective analysis, spanning over three decades, provides one of the most comprehensive datasets available on this topic. By demonstrating a clear benefit of Kock pouch conversion, it fills a critical gap in literature and offers practical guidance for clinicians managing patients with failed Barnett reservoirs.

The improved salvage rates observed in the conversion group are likely attributable to key differences in the valve mechanisms of the Barnett and Kock designs. The Barnett pouch relies on a collar valve mechanism, which, although innovative, has been prone to slippage, chronic leakage, and failure over time [2]. In contrast, the Kock pouch employs an intussuscepted nipple valve, created by invaginating the ileum and securing it with a stapled technique. This design is inherently more stable, reducing the likelihood of slippage and leakage. Furthermore, the use of a non-cutting TA stapler in constructing the new valve during conversion enhances structural integrity, offering a durable continence mechanism [1, 8]. The conversion procedure itself, while technically complex, preserves the original Barnett reservoir walls, saves bowel length by avoiding construction of an entire new pouch, and focuses on replacing the malfunctioning valve with a more reliable alternative. This targeted surgical approach minimizes the need for extensive reconstruction while addressing the primary source of pouch failure, thereby contributing to the higher salvage rates seen in the conversion group.

The technical complexity of Kock pouch conversion underscores the importance of surgical expertise in achieving successful outcomes. The procedure requires precise disconnection and reorientation of the afferent limb, careful resection of the collar valve, and meticulous construction of the new nipple valve [8]. These steps must be performed with precision to ensure that the converted pouch maintains its function over time. The success of this approach in the hands of experienced surgeons highlights the value of specialized training and the need for referral to high-volume centers with expertise in complex pelvic surgery [9, 10]. These findings align with prior research on continent ileostomy revisions, including a study comparing continent ileostomy conversion to redo ileal pouch–anal anastomosis (IPAA), which demonstrated favorable long-term outcomes for continent diversion. While that study focused on IPAA failure as an indication for conversion, our study specifically evaluates the transition from Barnett to Kock pouch, reinforcing the role of revisional continent ileostomy in achieving durable pouch salvage and avoiding permanent ileostomy [11]. Similar to studies on IPAA conversion to continent ileostomy, including recent work on strategic surgical considerations in pouch salvage, our findings highlight the feasibility of preserving the existing reservoir while reconstructing a more durable continence mechanism. This reinforces the role of revisional surgery in optimizing long-term outcomes and avoiding permanent ileostomy [12, 13].

Our study adds to the growing body of literature on continent ileostomy revisions, including the work by Duraes et al. that evaluated outcomes of redo continent ileostomies in patients with inflammatory bowel disease [14]. While their study analyzed a broader range of revision procedures across a larger cohort, our study focuses specifically on the conversion of Barnett pouches to Kock pouches, highlighting the technical advantages and long-term benefits of the stapled nipple valve design. Together, these studies underscore the importance of specialized surgical approaches in achieving durable pouch salvage and improving outcomes for patients with complex pouch failures.

The strengths of this study lie in its exceptionally long follow-up period, real-world data, and focus on a relatively rare and understudied procedure. The ability to track outcomes over a median of 159.5–301.6 months provides a robust perspective on the durability of Kock pouch conversions compared with non-conversion management. In addition, the inclusion of both functional and surgical outcomes ensures a comprehensive assessment of patient trajectories. Moreover, this study has several limitations. First, treatment allocation was not random. Conversion was not universally feasible and non-conversion often reflected unfavorable anatomy, frailty, or patient preference. In addition, this cohort represents a highly selected group of motivated patients, many of whom underwent initial continent ileostomy surgery decades ago—often 20–30 years prior—when K-pouch procedures were more common. These patients frequently had limited follow-up for long intervals before re-presenting with complications, and many struggled to find centers qualified to manage their pouch. As such, the observed differences between converted and non-converted groups likely reflect both treatment allocation and survivorship bias, as patients selected for conversion necessarily survived long enough and retained sufficient bowel function to become candidates for the procedure. These factors, together with unmeasured confounders such as comorbidity and surgical era, limit causal inference and support interpreting our findings as hypothesis-generating. Second, our findings are subject to survivorship bias, as patients in the conversion group necessarily survived and maintained sufficient pouch function to undergo reoperation, whereas some non-converted patients may have failed earlier or declined surgery. Third, the small sample size limits statistical power, resulting in wide confidence intervals and rendering our analyses exploratory rather than definitive. Fourth, the median follow-up after conversion was relatively short (31.5 months), which may not capture late complications or failures that occur over the decades-long disease course; thus, our results primarily reflect short- to mid-term outcomes. Finally, our definition of pouch failure was limited to structural endpoints (excision or permanent ileostomy) and did not incorporate patient-reported outcomes such as continence, function, or quality of life, potentially underestimating functional morbidity. Future studies with larger cohorts, longer follow-up, and integration of patient-centered outcomes are needed to validate and expand upon our findings.

Despite these limitations, the findings have significant clinical implications. Conversion of a failing Barnett pouch to a Kock pouch is a viable and effective strategy for avoiding permanent ileostomy in motivated patients. By addressing the underlying issue of valve failure with a more reliable design, the procedure offers a durable solution that can substantially improve quality of life. However, it is crucial to emphasize the need for careful patient selection, as this technically demanding surgery requires both the skill of an experienced surgeon and the commitment of patients to adhere to postoperative care and catheterization protocols. Given the favorable long-term outcomes demonstrated in this study, Kock pouch conversion should be considered for patients with failed Barnett reservoirs who are willing to undergo a technically complex procedure to retain continence.

In conclusion, this study demonstrates that conversion of a failed Barnett continent reservoir to a Kock pouch is feasible and associated with higher long-term pouch salvage and lower failure rates compared with non-conversion management. The procedure preserved bowel length and continence in most patients, confirming its effectiveness as a pouch-salvaging option in this cohort.

## Data Availability

The data generated during this study are available from the corresponding author upon reasonable request.
